# Assessing diversity and phylogeny of Indonesian breadfruit (*Artocarpus* spp.) using internal transcribed spacer (ITS) region and leaf morphology

**DOI:** 10.1186/s43141-023-00476-y

**Published:** 2023-02-09

**Authors:** Dindin Hidayatul Mursyidin, Akbar Setiawan

**Affiliations:** Laboratory of Genetics and Molecular Biology, Faculty of Mathematics and Natural Sciences, University of Lambung Mangkurat, Jl. A. Yani Km. 36, Banjarbaru, South Kalimantan 70714 Indonesia

**Keywords:** Genetic diversity, Breadfruit (*Artocarpus* spp.), Phylogenetic relationship, Breeding and preservation, Nuclear DNA

## Abstract

**Background:**

Breadfruit (*Artocarpus* spp.) is the main genus of Moraceae with multipurpose benefits, both ecologically and economically important, e.g., food ingredients, building materials, traditional medicine, and natural insecticides. However, most endemic *Artocarpus* have been threatened due to natural disasters and habitat degradation. The objective of our study was to determine the genetic diversity and relationships of endemic *Artocarpus* from South Borneo, Indonesia, using an internal transcribed spacer (ITS) region and leaf morphology.

**Results:**

Morphologically, endemic *Artocarpus* endemic to South Borneo, Indonesia, has a different leaf shape, i.e., narrow-obovate to broad-elliptic, from simple to deeply dissected. Following the ITS region, this germplasm has a moderate level of nucleotide diversity (0.069). The phylogenetic analysis revealed *Artocarpus* into four (4) main clades, where the nearest is shown by the ‘Puyian’ (*Artocarpus rigidus*) and ‘Binturung’ (*Artocarpus odoratissimus*) at a coefficient divergence of 0.027, whereas the furthest by ‘Kulur’ (*A. camansi*) and ‘Tiwadak’ (*A. integer*) at a coefficient of 0.132.

**Conclusion:**

In brief, although an endemic *Artocarpus* of South Borneo, Indonesia, has a moderate level of nucleotide diversity, this germplasm also shows a unique phylogenetic relationship. Thus, this information is urgent in supporting the future *Artocarpus* breeding and preservation programs, mainly to save this germplasm from being threatened.

**Supplementary Information:**

The online version contains supplementary material available at 10.1186/s43141-023-00476-y.

## Background

Breadfruit (*Artocarpus* spp.) is the main genus of Moraceae with multipurpose benefits, both ecologically and economically important, e.g., food ingredients, building materials, traditional medicine, and natural insecticides [1–3]. For example, *Artocarpus altilis* (‘sukun’— Java), *Artocarpus heterophyllus* (‘nangka’ — Java), *Artocarpus integer* (‘cempedak’ — Java), *Artocarpus lakoocha* (‘Mahat’ — Thai), *Artocarpus lanceifolius* (‘keledang’ — Banjar), and *Artocarpus kemando* (‘cempedak air’ — Malay) are six species of *Artocarpus* whose fruits are edible or can made for foodstuffs [4]. The bark of *A. lanceifolius*, *A. integer*, and *A. heterophyllus* can be used as building materials because they are permanent and durable [2]. The leaves of *A. altilis* are known to be efficacious in traditional medicine, especially for treating cirrhosis of the liver, hypertension, and diabetes [2].

Globally, *Artocarpus* has high genetic diversity, consisting of 70 species spread throughout the world, mainly in southern and southeastern Asia, including India, Sri Lanka, Pakistan, China, Malaysia, and Indonesia, as well as the Solomon Islands [1, 5]. For Indonesia, *Artocarpus* has recorded as many as 30 species and is present on five large islands, including Java, Sumatra, Borneo (Kalimantan), Sulawesi, and Maluku [6]. However, Borneo is the largest, with 23 species, and is estimated to be the center of the world’s *Artocarpus* diversity [1]. However, due to the deforestation or over-conversion of land, especially for agricultural, plantation, and settlement purposes, part of *Artocarpus* began to be scarce and difficult to find in the wild [7].

IUCN [8] reported seven *Artocarpus* with vulnerable status, i.e., *Artocarpus rubrovenus*, *Artocarpus anisophyllus*, *Artocarpus tamaran*, *Artocarpus treculianus*, *Artocarpus hypargyreus*, *Artocarpus nobilis*, and *Artocarpus blancoi*. Meanwhile, *A annulatus* and *A. nanchuanensis* are critically endangered [8]. Consequently, conservation or preservation, including their breeding and cultivation efforts to save various species of *Artocarpus*, is urgent. Conceptually, conservation activities aim to ensure the continuity of species, habitats, and biological communities, as well as interactions between species, including between species and their ecosystems [7]. Meanwhile, breeding and cultivation efforts aim to preserve essential genes with superior properties for future use [9].

On the other hand, the genetic characterization of germplasm using molecular markers is currently becoming alternative and complementary to the morphological characterization commonly used, as reported in *Artocarpus* by Jones et al. [10], Estalansa et al. [11], Karunarathne et al. [12], and Daley et al. [13]. In general, although quite expensive, molecular markers provide high speed and accuracy in the genetic characterization of germplasm [14]. In contrast, morphological ones tend to be time-consuming and are strongly influenced by environmental factors. Internal transcribed spacer (ITS) is the nuclear molecular marker that is useful in determining genetic diversity and the relationship of *Artocarpus*, including its taxonomy [5, 15].

Unlike chloroplast DNA with inherited maternally, the ITS is inherited biparentally [16]. Besides, due to the universality and simplicity of amplifying and showing a high mutation rate, the ITS provides better resolution in the estimation of the genetic diversity of most plants [16, 17]. Some plants that successfully verified using this marker are as follows: *Acanthopanacis* [18], *Anoectochilus* [17], *Dioscorea* [19], *Litsea* [20], *Uncaria* [21], and *Zanthoxylum* [22].

This study aimed to determine the genetic diversity and relationships of endemic *Artocarpus* to South Borneo, Indonesia, using ITS marker and leaf morphology. Our results provide essential information for future *Artocarpus* preservation and breeding tasks, especially in Indonesia.

## Materials and methods

### Plant materials

**Fig. 1:**
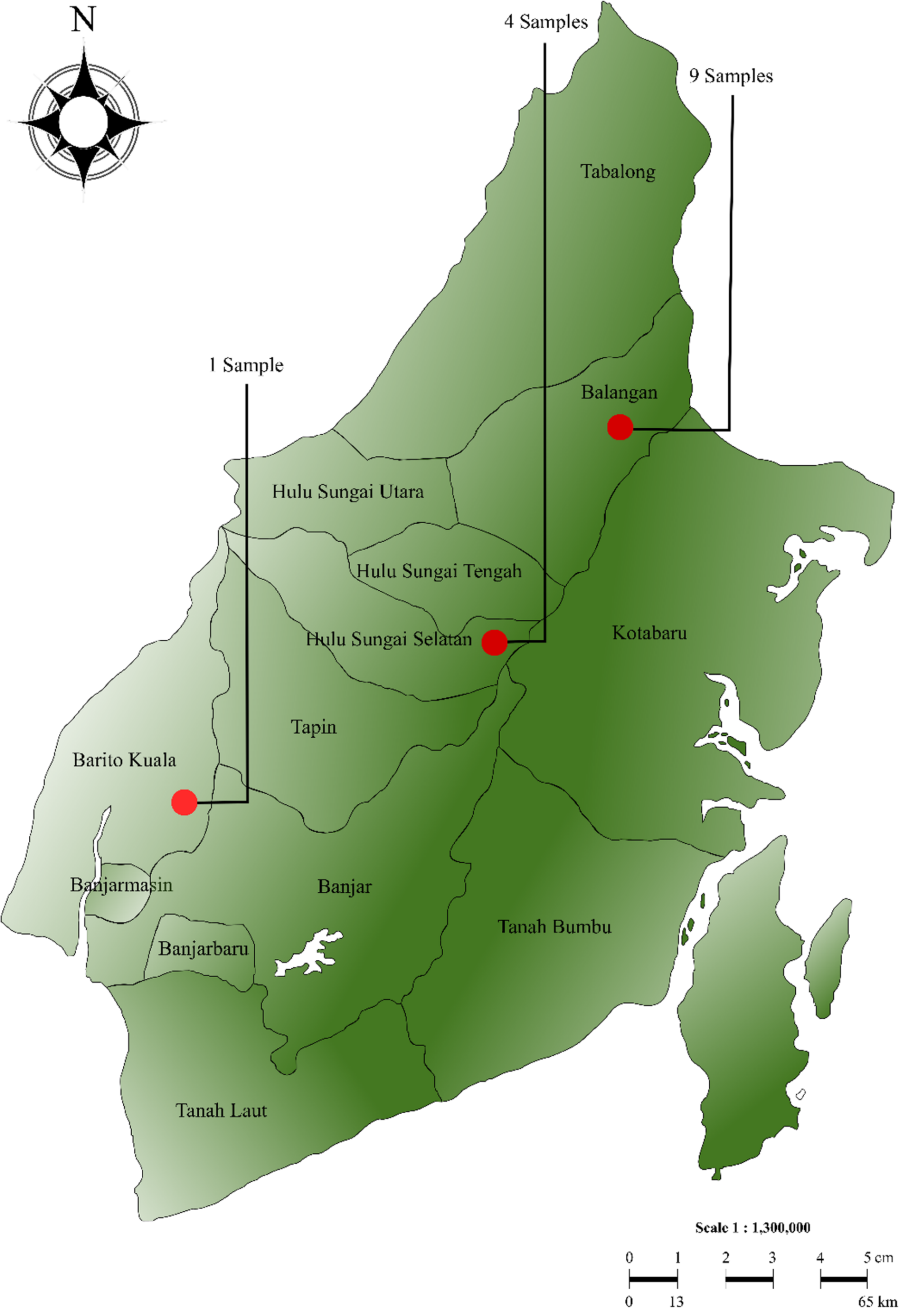
The map of South Borneo, Indonesia, shows the sampling location and the number of samples collected each. For more information about those samples, see Table 1

In this study, we used 14 (leaf) samples of breadfruit (*Artocarpus* spp.) collected from a part of Borneo Island, especially three regencies in South Borneo, Indonesia (Fig. 1). All were characterized morphologically and molecularly. Table 1 provides complete information about the *Artocarpus* samples used in the study.

**Table 1:** Breadfruit species (*Artocarpus* spp.) used in the study, including their origin and genetic status

Local name	Species	Code	Regency origin	Genetic status
‘Karusung’	*A. anisophyllus*	A1	Barito Kuala	Endemic
‘Tiwadak’	*A. integer*	A2	Balangan	Endemic
‘Kulur’	*A. camansi*	A3	Hulu Sungai Selatan	Endemic
‘Tarap’	*A. sericicarpus*	A4	Balangan	Endemic
‘Anjani’	*A. hirsutus*	A5	Hulu Sungai Selatan	Introduction^a^
‘Nangka’	*A. heterophyllus*	A6	Hulu Sungai Selatan	Endemic
‘Mantiwadaka’	*A. kemando*	A7	Balangan	Endemic
‘Kulidang’	*A. lanceifolius*	A8	Hulu Sungai Selatan	Endemic
‘Tampang Susu’	*A. limpato*	A9	Balangan	Endemic
‘Binturung 1’	*A. odoratissimus*	A10	Balangan	Endemic
‘Binturung 2’	*A. odoratissimus*	A11	Balangan	Endemic
‘Tampang’	*A. primackii*	A12	Balangan	Endemic
‘Puyian’	*A. rigidus*	A13	Balangan	Endemic
‘Tiwadak Banyu’	*A. teysmannii*	A14	Balangan	Endemic

Remarks: ^a^From India

### Morphological analysis

Morphological analysis was conducted by leaf characteristics only. It was performed descriptively using the guidance of Ragone and Wiseman [23].

### Molecular analysis

All leaf samples of *Artocarpus* were extracted by a commercial DNA extraction kit (GP100) from Geneaid Biotech Ltd., Taiwan. The concentration and purity of DNAs were determined by UV-VIS spectrophotometry. The DNAs were then amplified by ITS primers [24] (forward: 5′-TCGTAACAAGGTTTCCGTAGGTG-3′; reverse: 5′-TCCTCCGCTTATTGATATGC-3′), using these reactions: initial denaturation (94 °C for 5 m); denaturation (94 °C for 30 s); annealing (48 °C for 30 s); extension (72 °C for 45 s); and final extension (72 °C for 7 m) [25]. The PCR was employed using 25 μl of a total volume, comprising of 20 ng DNA template (2 μl), 0.2 μmol forward and reverse primers (0.5 μl each), and 22 μl of MyTaq HS Red PCR Mix (Bioline, UK). The PCR product (DNA target) was visualized by electrophoresis of agarose gel (2%) and a UV transilluminator and then sequenced bidirectionally using the Sanger method (1st Base Ltd., Malaysia).

### Data analysis

The data (ITS sequences of *Artocarpus*) were reconstructed and analyzed manually by the MEGA 11 [26]. All were then aligned using MultAlin [27] to find out some mutational events, both substitution (transition-transversion) and indels (insertion-deletion). The genetic diversity (*π*) was determined using the Nei and Li [28] criteria, i.e., low (≤ 0.04), moderate (0.05–0.07), and height (≥ 0.08), whereas haplotype (h) and haplotype diversity (Hd) by DnaSP ver. 6.0 [29]. The phylogenetic tree was reconstructed by the maximum likelihood (ML) with a Kimura-2 parameter and a nearest-neighbor-interchange (NNI) model [26, 30]. The phylogenetic tree was evaluated by bootstrap statistics (1000 replicates) and confirmed using principal component analysis or PCA [31]. The secondary structure ITS was also analyzed and reconstructed using the online software of ITS2 Workbench (http://its2.bioapps.biozentrum.uni-wuerzburg.de/) [32].

## Results

**Fig. 2:**
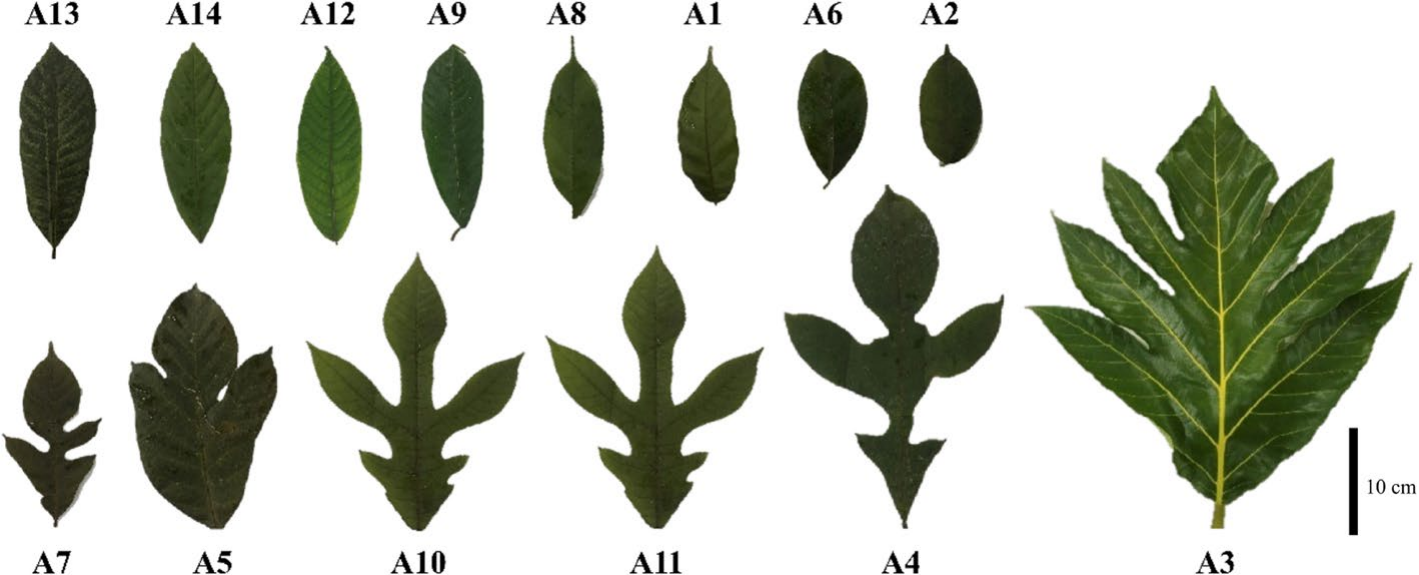
Leaf morphology of endemic breadfruit (*Artocarpus* spp.) from South Borneo, Indonesia, shows two different forms, i.e., narrow-obovate to broad-elliptic, from simple to shallowly dissected. The name of each sample is provided in Table 1

Morphologically, endemic *Artocarpus* has a different leaf shape, namely narrow-obovate to broad-elliptic, from simple to deeply dissected (Fig. 2). The simple narrow-obovate leaf was shown by ‘Tiwadak’ or *A. integer* (A2), whereas the broad-elliptic by ‘Puyian’ or *A. rigidus* (A13). Besides, the narrow deeply dissected leaf was shown by ‘Mantiwadaka’ or *A. kemando* (A7), whereas the broad one was by ‘Kulur’ or *Artocarpus camansi* (A3) (see Fig. 2).

**Fig. 3:**
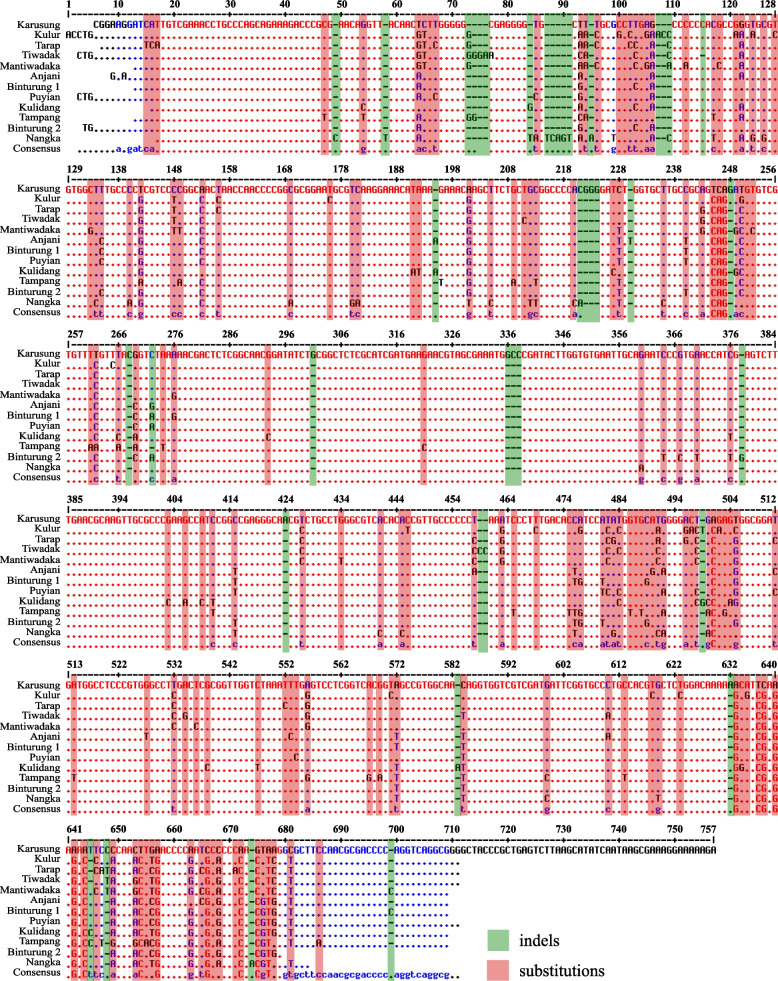
Multiple sequence alignments, showing two mutational events, i.e., substitutions and indels, from ITS sequence of endemic breadfruit (*Artocarpus* spp.) from South Borneo, Indonesia

**Fig. 4:**
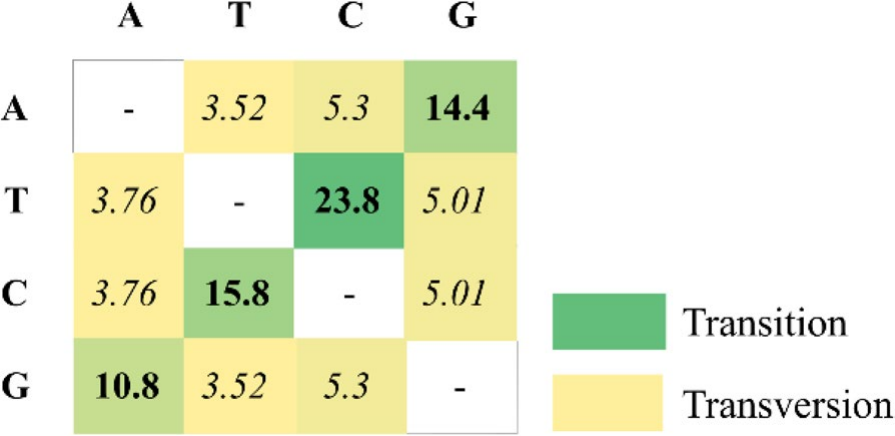
Nucleotide substitution patterns of endemic breadfruit (*Artocarpus* spp.) from South Borneo, Indonesia

**Fig. 5:**
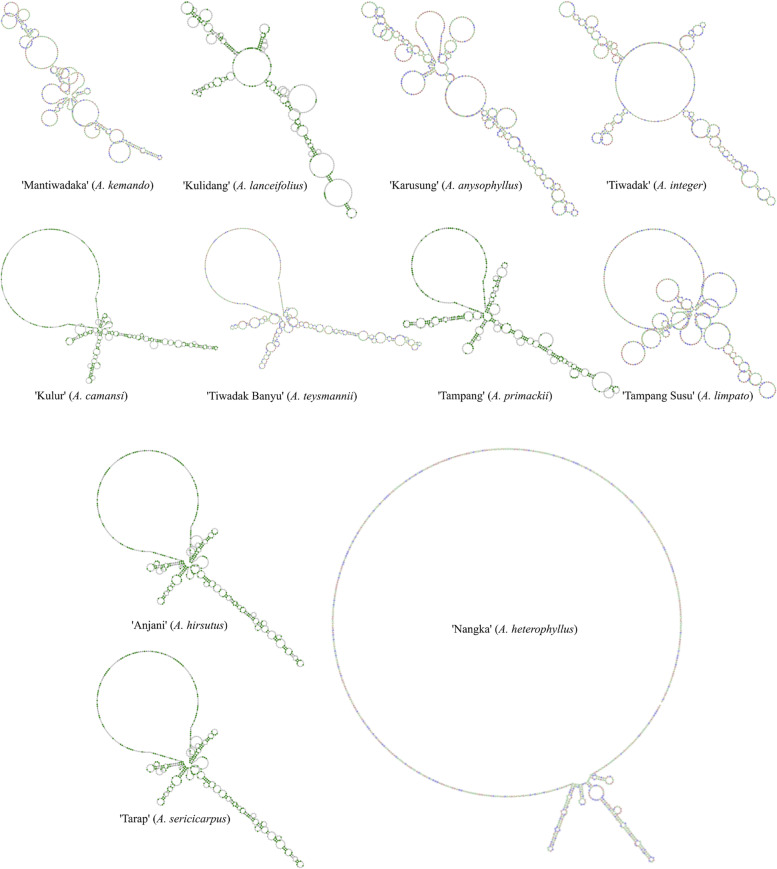
The secondary structures of the breadfruit (*Artocarpus* spp.) ITS sequence endemic to South Borneo, Indonesia, shows different forms. In this case, ‘Anjani’ (*A. hirsutus*) and ‘Tarap’ (*A. sericicarpus*) have similar

The sequencing results showed that endemic *Artocarpus* had different partial ITS sequence lengths, between 640 and 727 bp (Table 2). In this case, the shortest ITS sequence is shown by ‘Binturung-2’ (*A. odoratissimus*), whereas the longest is by ‘Karusung’ (*Artocarpus anysophyllus*). After being aligned, several mutation events are seen in the ITS sequence, especially substitutions and indels or insertions-deletions (Fig. 3). Table 3 presents various genetic characteristics of ITS *Artocarpus* sequences, from mutation events and GC content to nucleotide or molecular diversity. Meanwhile, Fig. 4 shows the substitution pattern of the ITS *Artocarpus* sequence in more detail. When reconstructed, these sequences have unique secondary structures (Fig. 5). However, only 11 of the 14 samples had predictable ones. Meanwhile, ‘Anjani’ (*Artocarpus hirsutus*) and ‘Tarap’ (*Artocarpus sericicarpus*) have a similar ITS secondary structure (see Fig. 5).

**Table 2:** Endemic breadfruit species (*Artocarpus* spp.) used in the study, including the length of ITS sequences

Local Name	Species	Code	ITS length (bp)
‘Karusung’	*A. anisophyllus*	A1	727^b^
‘Tiwadak’	*A. integer*	A2	643
‘Kulur’	*A. camansi*	A3	679
‘Tarap’	*A. sericicarpus*	A4	669
‘Anjani’	*A. hirsutus*	A5	665
‘Nangka’	*A. heterophyllus*	A6	697
‘Mantiwadaka’	*A. kemando*	A7	663
‘Kulidang’	*A. lanceifolius*	A8	661
‘Tampang Susu’	*A. limpato*	A9	660
‘Binturung 1’	*A. odoratissimus*	A10	660
‘Binturung 2’	*A. odoratissimus*	A11	640^a^
‘Tampang’	*A. primackii*	A12	660
‘Puyian’	*A. rigidus*	A13	671
‘Tiwadak Banyu’	*A. teysmannii*	A14	677

Remarks: ^a^Shortest, ^b^longest

**Table 3:** The genetic information of ITS sequence of endemic breadfruit (*Artocarpus* spp.) from South Borneo, Indonesia^a^

Parameter	ITS
Number of observed bases (*n*)	760
Polymorphic (segregating) sites (bp)	215
Substitution (transition-transversion) sites (bp)	174
Indel (insertion-deletion) sites (bp)	53
Singleton (single-nucleotide polymorphism) sites (bp)	110
Parsimony informative sites (bp)	89
Nucleotide diversity (π)	0.069
Number of haplotypes (h)	14
Haplotype (gene) diversity (Hd)	1.000
GC content (%)	58.64
Bayesian information criterion (BIC)	5992.844
Akaike information criterion (AICc)	5807.212
Maximum likelihood value (ln*L*)	−2877.531

^a^By Kimura 2 (K2) model

Following Fig. 3 and Table 3, the total mutation or polymorphic (segregating) site of *Artocarpus* ITS is 215 bp, of which substitution (174 bp) is more than indels (53 bp). In addition, singleton mutations (110 bp) are also higher than parsimony informative sites (89 bp) (see Table 3). Referring to the similar table, *Artocarpus* has a nucleotide diversity of 0.069, a haplotype diversity of 1.000, with a GC content of 58.64%. Furthermore, based on Fig. 4, the ITS *Artocarpus* sequence has the highest substitution pattern for the transition of 23.8 for the change of T to C, while the transversion is 5.3 for G to C and C to A, respectively.

**Fig. 6:**
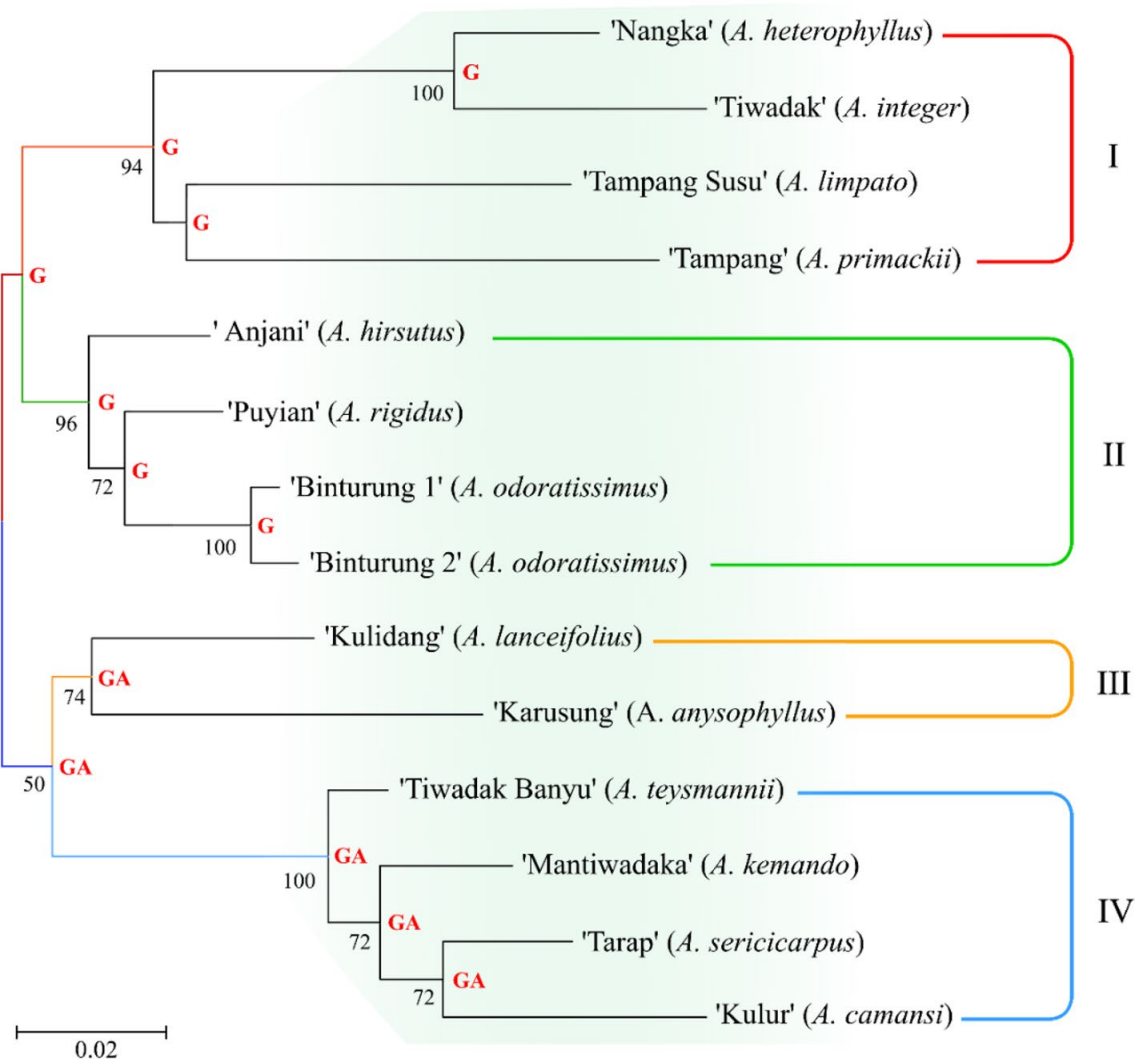
The phylogenetic tree shows the grouping of endemic breadfruit (*Artocarpus* spp.) from South Borneo, Indonesia, into four main clades. Values and letters (nucleotides) on nodes indicate the bootstrap analysis 1000 times and possible ancestors, respectively

**Fig. 7:**
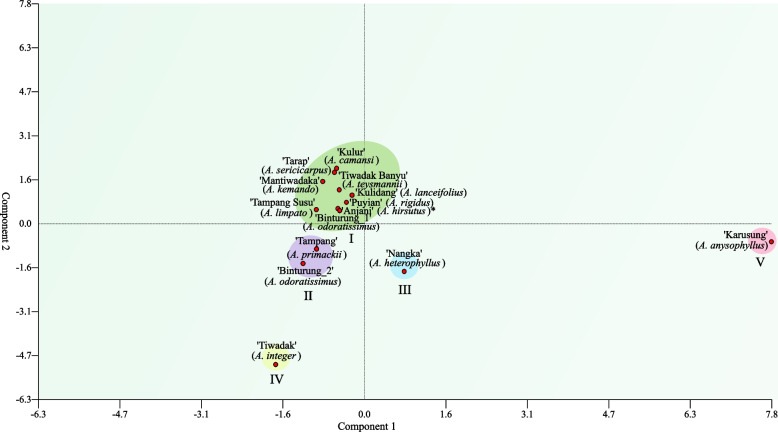
Grouping of endemic breadfruit (*Artocarpus* spp.) from South Borneo, Indonesia, into five main groups based on principal component analysis (PCA)

Analysis of genetic relationship used the maximum likelihood method, separating endemic *Artocarpus* into four (4) main clades, of which three clades (I, II, and IV) consist of 4 samples or members while the remaining (clade III) are only two (Fig. 6). However, the principal component (PCA) analysis confirmed the grouping of *Artocarpus* into five groups, where the first was the largest with nine samples (Fig. 7).

Based on Fig. 6, *Artocarpus* was split into two large groups polyphyletically, of which clades 1 and 2 became the first group with G ancestor, while clades 3 and 4 were the second groups (GA ancestor). In other words, the first group, consisting of clades 1 and 2, is monophyletic, as are clades 3 and 4, because they have the same ancestry, respectively. In this case, ‘Tiwadak’ or breadfruit (*A. integer*) has the closest relationship to ‘Nangka’ or jackfruit (*A. heterophyllus*), similarly ‘Tampang Susu’ (*A. limpato*) with ‘Tampang’ (*A. primackii*) (see Fig. 6).

**Fig. 8:**
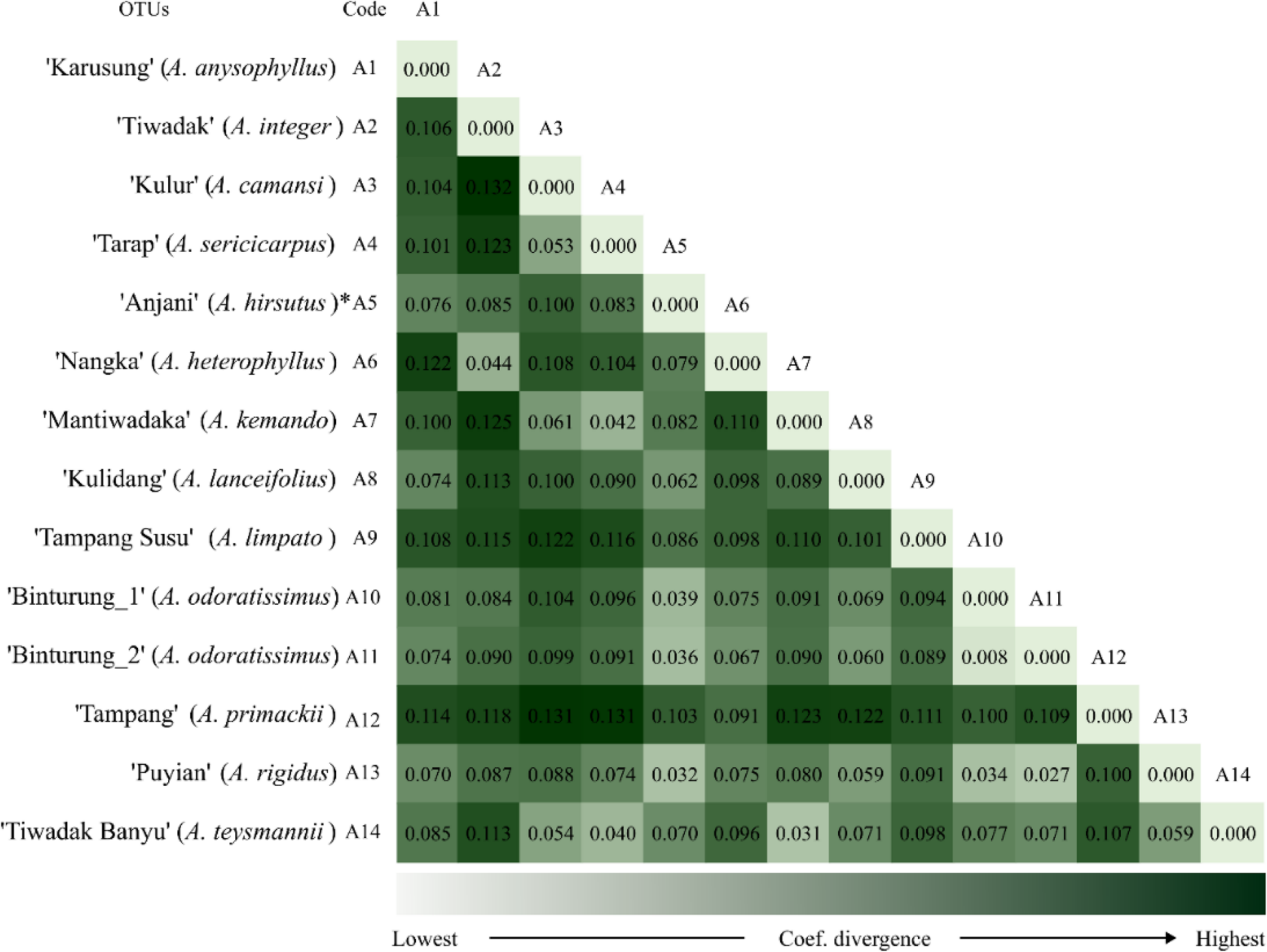
The heatmap shows the relationship among endemic breadfruit (*Artocarpus* spp.) from South Borneo, Indonesia, based on the coefficient divergence value. In this case, the lower the coefficient value, the closer the genetic relationship would be

Further analysis (Fig. 8) shows that the relationship of the nearest *Artocarpus* belongs to the ‘Puyian’ (*A. rigidus*) with the ‘Binturung_2’ (*A. odoratissimus*) at a coefficient divergence of 0.027. In contrast, the furthest relationship is between ‘Kulur’ (*A. camansi*) and ‘Tiwadak’ (*A. integer*) at a coefficient of 0.132 (Fig. 8). In addition, the relationship of ‘Tiwadak’ (*A. integer*) with ‘Jackfruit’ (*A. heterophyllus*) is recorded at coefficient 0.044, as for ‘Tampang Susu’ (*A. limpato*) and ‘Tampang’ (*A. primackii*) at 0.111 (see Fig. 8).

## Discussion

Based on morphological observations, endemic breadfruit (*Artocarpus* spp.) from South Borneo, Indonesia, shows a different leaf shape, i.e., simple narrow-obovate to broad-elliptic deeply dissected (Fig. 2). According to Doyle [33], simple leaves exhibit primitive evolution rather than more complex dissected. It corresponds to the ‘telome’ theory, which leaves are proposed to have evolved through independent processes of branching, planation, webbing, and overtopping [34]. Referring to Harrison and Morris [35], initial constraints to leaf evolution probably involved several factors, e.g., high atmospheric global temperatures, low stomatal densities, and low water uptake capacities before root evolution.

Following the Nei and Li [28] criteria, this germplasm has a moderate level of genetic diversity (see Table 3). It is quite an encouraging thing. In plant breeding activities, information about genetic diversity is indispensable. According to Witherup et al. [36], maintaining crop diversity is urgent in retaining their capability for future adaptation. In other words, insufficient genetic diversity may cause crop failures. Also, maintaining genetic diversity is essential to support the current and upcoming crop breeding initiatives.

In concept, mutations are the main factor giving rise to genetic diversity. In other words, mutations are genetic changes that occur spontaneously in an allele or chromosome in which genetic diversity appears [37]. In this study, the ITS sequence of *Artocarpus* showed several mutations, especially substitutions and indels (see Fig. 3). According to Chen and Shiau [38], this region has a higher nucleotide substitution rate, making it suitable for genetic diversity and the related study between germplasm [19]. Besides, the gene occasionally exhibits entire codon insertions or deletions, showing a conservative pattern of nucleotide replacement [39]. However, although this mutation has significant benefits remains the question in some plant families of whether a gene can maintain the stable structure and function of the protein produced [40].

Apart from the presence of mutations in the ITS sequence of *Artocarpus*, using wild or underutilized populations (minor crops) with certain important traits may broaden the genetic diversity level and improve agricultural quality for both pests and diseases resistance; drought, salinity, and other abiotic stresses tolerance; and gain higher quality and yield ability. Witherup et al. [36] also state that most underutilized or minor crops provide a unique opportunity to quantify genetic diversity baselines before bottleneck selection is present and to implement practice learning from other crops that have previously suffered losses. Although most studies focus on the diversity loss of primary crops, many minor crops are now systematic improvement targets for the development of uniform lines and industrialization [9].

In particular, the development of *Artocarpus* is currently directed toward the dwarf cultivars assembling. For example, breadfruit (*A. altilis*), a traditional staple crop for starchy in the tropics, is modified to be dwarf due to vulnerability to wind damage [40]. In this case, dwarfing rootstocks of breadfruit may come from wild relatives, for example, *A. anisophyllus*, *A. hirsutus*, *A. camansi*, *A. nitidus*, *Artocarpus mariannensis*, *A. integer*, *A. heterophyllus*, *A. lakoocha*, *Artocarpus petelotii*, and *Artocarpus xanthocarpus*. The last two species may be good candidates for dwarfism because they have a height of about 10 and 8 m, respectively [41].

Thus, information about genetic relationships is also essential to consider. In this study, the nearest *Artocarpus* is shown by the ‘Puyian’ (*A. rigidus*) and ‘Binturung’ (*A. odoratissimus*) at a coefficient divergence of 0.027, whereas the furthest by ‘Kulur’ (*A. camansi*) and ‘Tiwadak’ (*A. integer*) at a coefficient of 0.132 (Fig. 8). According to Mursyidin and Makruf [7], *Artocarpus* exhibits a complex phylogenetic relationship. For example, using nucleus genes, Zerega and Gardner [3] reported the close relationship between *Artocarpus nitidus*, *A. heterophyllus*, and *A. camansi*. Following microsatellite markers, Zerega et al. [36] revealed the closeness of *A. camansi* to *A. mariannensis*. Meanwhile, based on the complete chloroplast gene, Li and Song [42] reported the genetic proximity between *A. heterophyllus* and *Artocarpus nanchuanensis*.

According to Acquaah [43], when two individuals with far relationships cross, the genetic diversity of the offspring may be extant. Conversely, if the two closely related crosses, it will produce offspring with narrow genetic diversity. However, for the latter case, crosses between such elders tend to be avoided as they can result in inbreeding descendants. Referring to de Los Reyes [44], inbreeding will increase susceptibility to disease and stress, including decreasing crop yields.

## Conclusion

The results showed that breadfruit (*Artocarpus* spp.) from South Borneo, Indonesia, has two types of leaf morphology with moderate genetic diversity at the nucleotide level, amounting to 0.069. Meanwhile, the genetic relationship analysis revealed that the nearest *Artocarpus* is shown by the ‘Puyian’ (*A. rigidus*) and ‘Binturung’ (*A. odoratissimus*) at a coefficient divergence of 0.027, whereas the furthest by ‘Kulur’ (*A. camansi*) and ‘Tiwadak’ (*A. integer*) at a coefficient of 0.132. This information is urgent in supporting the future *Artocarpus* breeding and preservation programs.

## Supplementary Information

**Additional file 1. **The ITS transcript of each *Artocarpus* sample used in this study.

## Data Availability

All samples of endemic breadfruit (*Artocarpus* spp.) used in this study were deposited in the Laboratory of Genetics and Molecular Biology, Faculty of Mathematics and Natural Sciences, University of Lambung Mangkurat, Indonesia.

## Abbreviations

ITS: Internal transcribed spacer
IUCN: The International Union for Conservation of Nature
ML: Maximum likelihood
PCA: Principal component analysis
PCR: Polymerase chain reaction
