# Supplementary material for: Assessing diversity and phylogeny of Indonesian breadfruit (Artocarpus spp.) using internal transcribed spacer (ITS) region and leaf morphology
Source: J Genet Eng Biotechnol. 2023 Feb 9;21:15. doi: 10.1186/s43141-023-00476-y (PMC9911577; doi:10.1186/s43141-023-00476-y)

**Supplementary materials**

The ITS transcript of each *Artocarpus* sample used in this study

>Karusung (*A. anysophyllus*) 727 bp


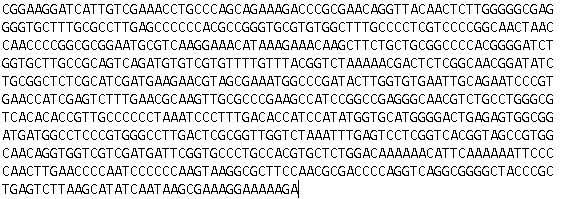


>Tiwadak (*A. integer*) 643 bp


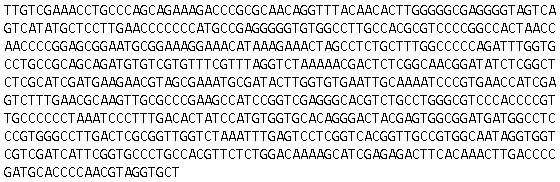


>Kulur (*A. camansi*) 679 bp


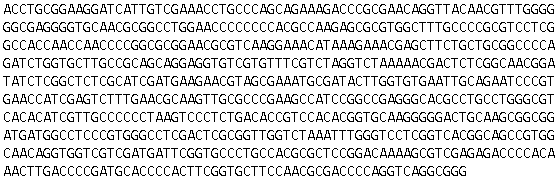


>Tarap (*A. sericicarpus*) 669 bp


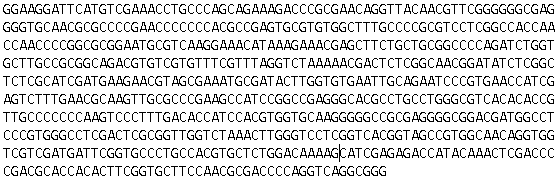


>Anjani* (*A. hirsutus*) 665 bp


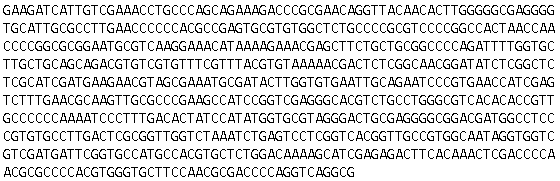


>Nangka (*A. heterophyllus*) 628 bp


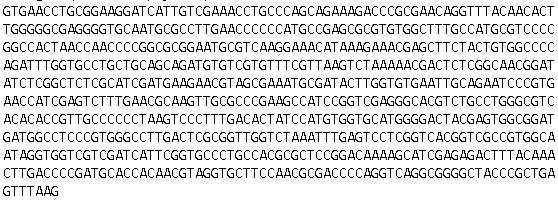


>Mantiwadaka (*A. kemando*) 663 bp


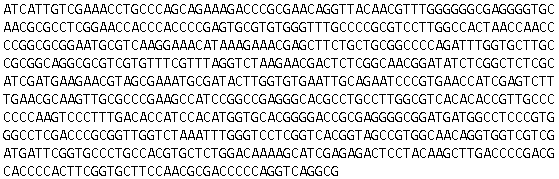


>Kulidang (*A. lanceifolius*) 661 bp


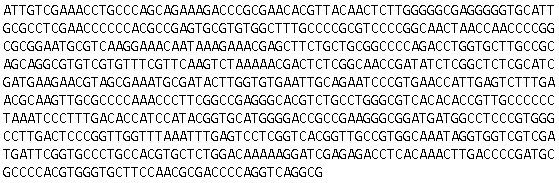


>Tampang Susu (*A. limpato*) 660 bp


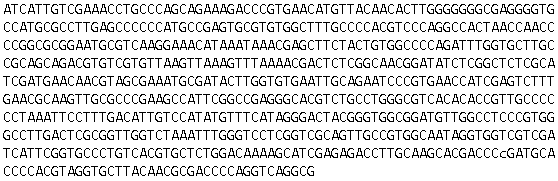


>Binturung_1 (*A. odoratissimus*) 660 bp


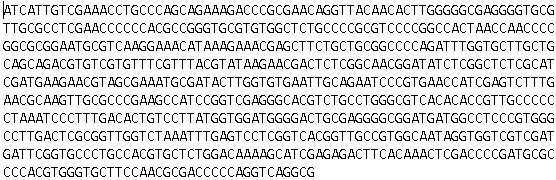


>Binturung_2 (*A. odoratissimus*) 640 bp


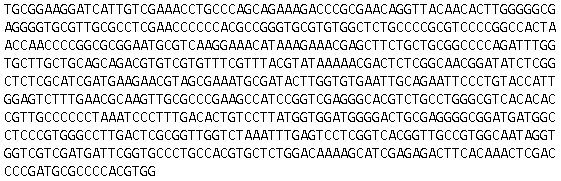


>Tampang (*A. primackii*) 660 bp


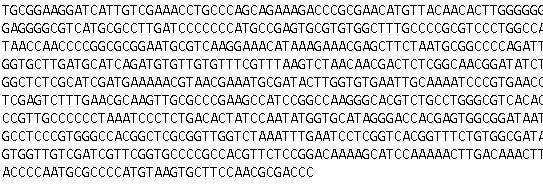


>Puyian (*A. rigidus*) 671 bp


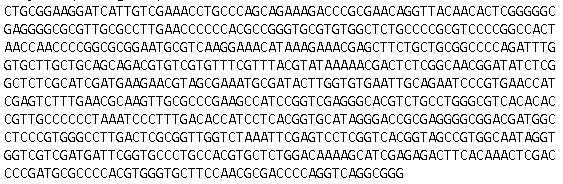


>Tiwadak Banyu (*A. teysmannii*) 677 bp


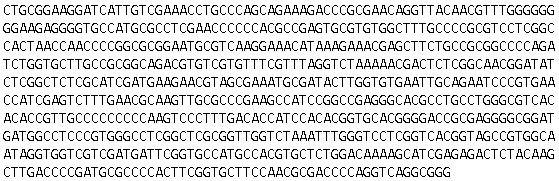

Supplement: Supplementary file 1 — Additional file 1. The ITS transcript of each Artocarpus sample used in this study. [file 43141_2023_476_MOESM1_ESM.docx]
